# Estimating the minimum important difference in the ALSFRS-R-instrument in people living with MND

**DOI:** 10.1080/21678421.2024.2447916

**Published:** 2025-02-03

**Authors:** Sarah L. Boddy, Rebecca M. Simpson, Stephen J. Walters, Hannah Bamford, Theresa Walsh, Christopher J. McDermott, PROSEC3 STUDY GROUP

**Affiliations:** School of Medicine and Population Health, The University of Sheffield, Sheffield, UK; 2 School of Medicine and Population Health, Sheffield Teaching Hospitals NHS Foundation Trust, UK; 3 Lancashire Teaching Hospitals NHS Foundation Trust, UK; 4 Oxford University Hospitals NHS Foundation Trust, UK; 5 West Suffolk NHS Foundation Trust, UK; 6 Hywel Dda University Health Board, UK; 7 NHS Lothian, Scotland; 8 Northern Care Alliance NHS Foundation Trust, England; 9 University Hospitals of North Midlands NHS Trust, England; 10 Newcastle Hospitals NHS Foundation Trust, England; 11 University Hospitals Coventry and Warwickshire, England; 12 St Joseph’s Hospital Auburn, Australia; 13 NHS Tayside, Dundee, Scotland; 14 The Walton Centre NHS Foundation Trust, Liverpool, England; 15 Phyllis Tuckwell Hospice Care, UK; 16 Sussex Community NHS Foundation Trust, UK; 17 St George's University Hospitals NHS Foundation Trust, London, England; 18 King’s College Hospital NHS Foundation Trust, London, England; 19 Cambridge University Hospitals NHS Foundation Trust, Cambridge, England; 20 University Hospitals Plymouth NHS Trust, England; 21 Keech Hospice Care, Luton, UK; 22 Medway Community Healthcare, UK; 23 LOROS, Hospice care for Leicester, Leicestershire and Rutland, Leicester, England; 24 Somerset Partnership NHS Foundation Trust, England; 25 Royal Free London NHS Foundation Trust, London, England; 26 Leeds Community Healthcare NHS Trust, Leeds, England; 27 St Margaret's Somerset Hospice, UK; 28 Manchester University NHS Foundation Trust, UK; 29 Birmingham Hospice (formerly St Mary’s Hospice), Birmingham, UK; 30 Mary Stevens Hospice, Stourbridge, UK; 31 Marie Curie Hospice, Bradford, UK; 32 Marie Curie Hospice, Solihull, UK; 33 NHS Ayrshire and Arran, Crosshouse, Scotland

**Keywords:** Motor neuron disease, MND, amyotrophic lateral sclerosis, ALS, ALSFRS-R, functional rating scale, minimal important difference, MID

## Abstract

*Objective*: The Amyotrophic Lateral Sclerosis Functional Rating Scale (ALSFRS-R) is a commonly used outcome measure in clinical trials for motor neuron disease (MND) therapies. As such, understanding how differences in scores relate to patient perception of their disease status is important when interpreting ALSFRS-R data. Our study sought to estimate the minimal important difference (MID) for the ALSFRS-R, the smallest difference in scores at which patients perceive a change in their quality of life. *Methods*: Data were collected as part of a longitudinal, observational saliva management study, ProSec3. These included both the ALSFRS-R and a global rating of change question (GRoC), which asked participants to rate how their disease had progressed since the previous visit. Anchor-based and distribution-based methods have been used to estimate the MID of the ALSFRS-R. The MID was estimated using two methods of calculating the total ALSFRS-R score, the original summation scale method and the recently proposed interval scale method. *Results*: A total of 145 people with MND had longitudinal ALSFRS-R and GRoC data. Different methods estimated the ALSFRS-R MID to be in the range of 2.02–5.43 for the summation scale and 1.23–3.31 for the interval scale method over a 3-month period, the time between study visits. Using anchor-based methods our MID estimates for the ALSFRS-R are 3.8 points and 2 points, respectively. *Conclusions*: The results of this study can guide clinicians and researchers in the interpretation of ALSFRS-R data. However, further studies are required to more precisely estimate the ALSFRS-R MID.

## Introduction

With prevalence estimated between 4.1 and 8.4 per 100,000 (1), Amyotrophic Lateral Sclerosis (ALS) represents a significant clinical burden to healthcare providers worldwide. Whilst a cure remains elusive, the number of trials of potential therapies aimed at slowing disease progression is growing. To assess efficacy, validated instruments have been developed to evaluate physical function in patients with ALS including the revised ALS functional rating scale (ALSFRS-R).

The ALSFRS-R is a measure designed to enable the assessment of physical functioning in people living with motor neuron disease (plwMND) (2). It is a 12-item validated instrument scored on a 0–48 scale consisting of four functional subdomains of equal weighting (bulbar, fine motor, gross motor, respiratory). A score of 48 represents normal function whilst 0 represents the worst performance across each of the measured dimensions. The ALSFRS-R was designed to be conducted via interview in-person, but has demonstrated reliability when undertaken over the telephone (3), via videoconferencing (4), and via adapted self-complete versions (5,6).

Despite its widespread use as an outcome measure in clinical trials, the utility of the ALSFRS-R has been criticized. Studies have demonstrated the total score does not decline linearly or accurately represent between-patient differences (7–9). Suggestions have been made regarding how the ALSFRS-R might be better used to model disease progression (7,10), but simple summation of score remains the most commonly employed method. The ALSFRS-R is often analyzed as an interval measure despite being categorical. For a scale to be considered interval, the difference between two values must be meaningful and consistent. As the ALSFRS-R is non-linear, declines of the same number of points are not equivalent. Recently, a method of transforming the raw score to an interval scale using bifactor Rasch Analysis has been proposed (11). This method attempted to identify variance common across the subdomains and discard any that was specific to each. This approach removes bias and reduces errors in the interpretation of disease decline.

Whilst it is relatively simple to determine the statistical significance of a change in function as defined by declining ALSFRS-R score, placing the magnitude of these changes in a context that is meaningful for patients, health professionals and other stakeholders is not so easy. Determination of the minimal important difference of the ALSFRS-R would help to address this problem.

The minimal important difference (MID) has been defined as, *‘The smallest difference in score in the domain of interest which patients perceive as beneficial and which would mandate, in the absence of troublesome side effects and excessive cost, a change in the patient’s management’* (12). In the context of the ALSFRS-R, the MID would represent a minimum difference at which a patient notes a corresponding change in quality of life (QoL).

The ALSFRS-R MID needs to be defined before its results can be interpreted meaningfully when assessing the benefits of new treatments for ALS. We have sought to estimate the MID of the ALSFRS-R using both anchor-based and distribution-based methods (13).

The aim of this study was to estimate the MID for the ALSFRS-R for comparisons between groups of people with MND using data from the existing ProSec3 study. This was done for both the original simple summation score method and the newly proposed transformation onto interval scale method.

## Materials and methods

### The ProSec3 study

Data were collected as part of ProSec3, a study exploring secretion management in plwMND, between February 2018 and September 2020. ProSec3 included 34 sites from across the UK and one in Australia, and collected data from 479 participants. Participants were eligible to take part if they were: attending MND clinics at a participating site, over 18 years of age, had a diagnosis of MND (ALS, primary lateral sclerosis, progressive muscular atrophy) and had capacity to give consent to take part. Data were collected at approximately 3-month intervals, aligned with regular clinic visits and included the ALSFRS-R and a global rating of change question (GRoC). The study was approved by the South Central-Hampshire B Research Ethics Committee (ref: 18/SC/0031) and informed consent was a requirement for participation.

### Instruments and scoring

The ALSFRS-R score was calculated using both the original simple summation score and the newly proposed transformation scale. For the summation method, the 12 questions were summed over the four domains to give a total ALSFRS-R score ranging from 0 to 48 with a higher score indicating better function/absence of symptoms. The revised method uses interval level transformation based on the raw score with the same 0–48 scale range overall (11). Summation scores were converted to interval scores as indicated in table 3 of the original study (11). As there is no set process for dealing with missing data for the ALSFRS-R, we have used a within-person – within-domain mean imputation. Within each of the four domains, if at least 2 of the 3 questions were answered, then mean imputation was used. The method used for missing data resulted in some patients receiving a total score which included a decimal, hence, their score was rounded up so the transformation to the interval scale could be applied. ENCALS guidelines were followed when conducting ALSFRS-R interviews, with the vast majority of data collected via interview and a small subset (*n* = 15) completed using a self-complete version.

The GRoC asked the participant “Since the last time you saw us, has there been any change in your overall health related quality of life?,” with three responses: “worse,” “about the same” and “better.” The GRoC was completed by the participant, with assistance where requested. GRoC data were collected alongside the ALSFRS-R, but only for the latter part of the study. The GRoC was implemented to the ProSec3 study in May 2019, 15 months after the start of the study. Therefore, the number of participants with relevant responses to the GRoC is considerably lower (*n*= 145) than those who had answered the ALSFRS-R questionnaire (*n* = 455), which was part of the study from its outset.

### Statistical analysis

All analyses were conducted using R version 3.6.0 (14).

Summary statistics are used to describe the demographic data for the ProSec3 population (*N* = 479) and for the population who answered the GRoC (*n* = 145). Participants were categorized into fast and slow progressors, with a decline rate in ALSFRS-R of ≥ 0.9 points or < 0.9 points per month respectively. This score was calculated preslope, assuming the patients had a score of 48 (max) at their symptom onset date.

To calculate disease progression using the ALSFRS-R questionnaire, the mean score for the full population was calculated at each of the five study timepoints. This data was plotted to visualize the natural decline. To estimate the decline, a random effects linear regression model was fitted with a random intercept and slope, and a continuous fixed variable for time coded as 0 months to 12 months for each of the five visits.

Both anchor-based and distribution-based methods were used to determine the MID for the ALSFRS-R as recommended by Revicki et al. (13). For the anchor-based methods, the GRoC was used at the second visit. This GRoC was considered as an anchor for ALSFRS-R as it is a self-reported single question rating of overall quality of life. Change in ALSFRS-R scores between that and the previous visit were grouped by GRoC response. Mean ALSFRS-R scores at each visit (visits 1 and 2) were calculated for each group.

Spearman’s correlation coefficient was calculated to assess the correlation between the ALSFRS-R and the GRoC anchor with values over 0.3 deemed as having acceptable association between anchor and the outcome measure (13).

Distribution-based methods are used as supporting evidence for the MID as they are sample dependent. The standardized effect size was calculated using the between-person standard deviation (SD) at baseline from the ProSec3 study data for small (0.2 SD) to medium (0.5 SD) effect sizes (15). We have used the SD yielded from the full dataset (rather than the GRoC subsample) to estimate the distribution-based effect sizes.

The standard error of measurement (SEM) is used to estimate the minimum detectable change (MDC) the variation in an individual’s “true score” when repeated measures have been undertaken (16). The MDC or minimal detectable difference “*is the smallest change in score that can be detected after allowing for ‘measurement errors’ or random errors*.” (17). The SEM is not used to calculate the MID but can be used as a proxy alongside other methods (16). The MID and MDC are important but they are different concepts measuring different things. The MDC is a statistical property of the measurement, but the MID is the value of concern for interpretation and is based on the judgment of patients (18,19).

To calculate the SEM, a value of 0.71 for internal consistency (Cronbach’s alpha) was used to determine reliability. This value was taken from the work by Cedarbaum et al. (2). For the re-test reliability (ICC), a value of 0.95 was taken from the work by Kaufmann et al. (3). To calculate SEM the following formula was used:

SEM = SD ×√(1−r)

where *r* is the reliability estimate and SD is the standard deviation.

## Results

### Demographics

Table 1 summarizes the characteristics of the cohort. The majority of participants had a diagnosis of ALS (ProSec3 81.8%, GRoC 80.7%), with a larger proportion reporting limb-onset than bulbar (ProSec3 67.6%, GRoC 65.5%). The cohort was male-biased (ProSec3 61.6%, GRoC 60.0%).

**Table 1.** Characteristics of the study population.

	ProSec 3 population (*N* = 479)		Those with GRoC population (*N* = 145)	
Characteristic	Mean (SD)	Median (IQR)	Mean (SD)	Median (IQR)
Age	64.5 (10.9)	66.0 (57.0–73.0)	64.0 (10.0)	64.0 (58.0–71.0)
	*N*	%	*N*	%
Sex				
Male	295	61.6	87	60.0
Female	176	36.7	54	37.2
Missing	8	1.7	4	2.8
MND Onset type				
Familial	30	6.3	11	7.6
Sporadic	443	92.5	133	91.7
Missing	6	1.3	1	0.7
MND Onset site				
Bulbar	127	26.5	44	30.3
Limb	324	67.6	95	65.5
Other	25	5.2	6	4.1
Missing	3	0.6	0	0
MND diagnosis criteria				
ALS	392	81.8	117	80.7
Not ALS (other MND diagnosis)	81	16.9	26	17.9
MND (unspecified)	6	1.2	2	1.4
Progression (summation scale)				
Fast	98	20.5	25	17.2
Slow	353	73.7	119	82.1
Missing	28	5.8	1	0.7
Progression (interval scale)				
Fast	164	34.2	47	32.6
Slow	280	58.5	97	67.4
Missing	35	7.3	1	0.7

*Note*: The ProSec3 population includes all participants from the saliva management study, the GRoC population refers to the subset of those participants who had completed the overall health related GRoC at their second study visit necessary for the anchor-based MID estimate method referred to in the methods.

### Natural progression

**Figure 1.**
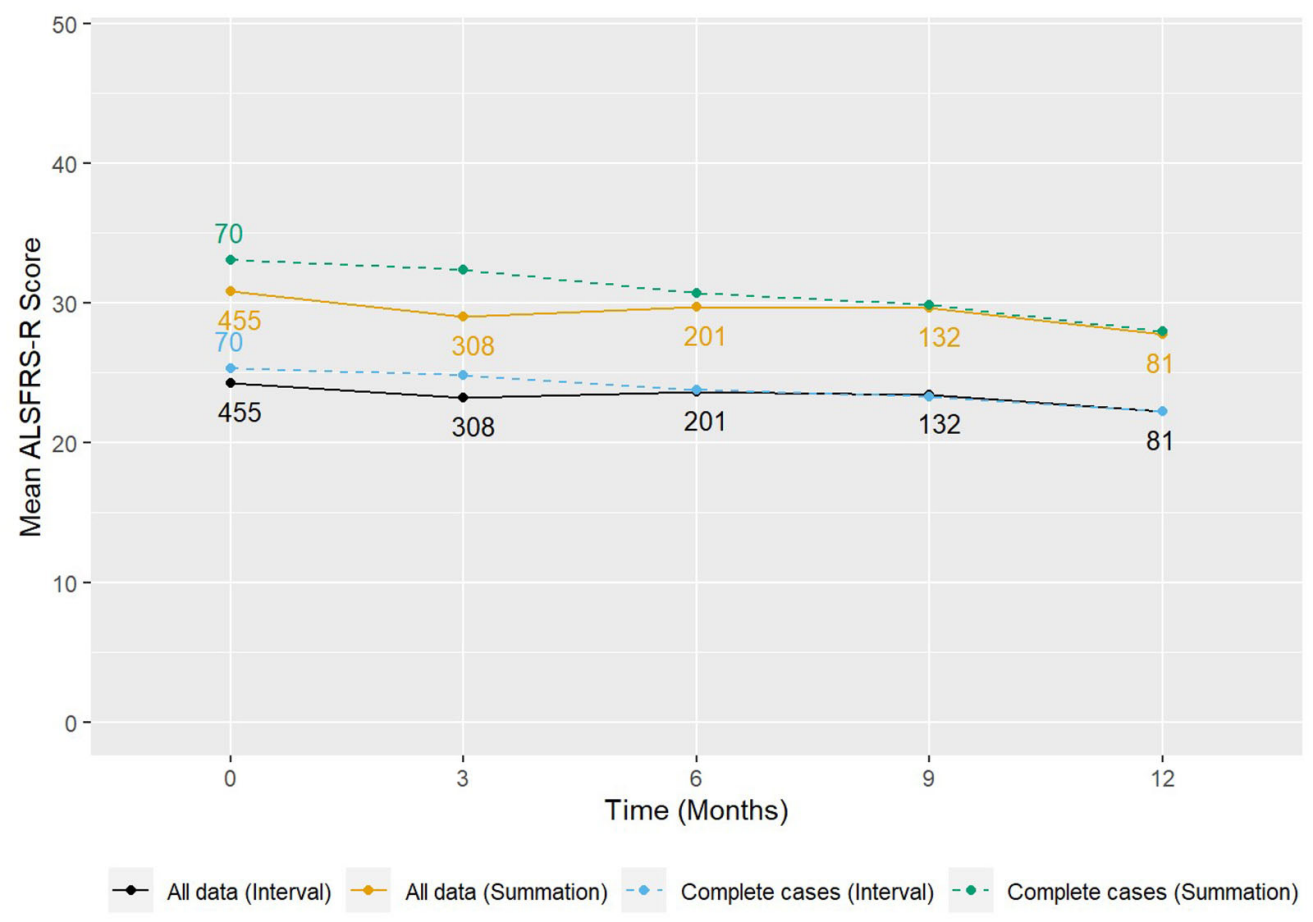
Natural progression of the ALSFRS-R scores across the five study visits. The continuous lines include all data (regardless of the number of visits individual participants completed), the broken lines only include participants who completed 5 study visits (though ALSFRS-R data may not be complete for all visits). Black and blue lines refer to transformed (interval) scores, green and orange lines are based on standard summation of question scores. Mean values are heavily influenced at the different visits by people with slow disease progression as people with fast-progressing MND were less likely to complete later visits. *Mean ALSFRS-R score over time split by all data and those with complete cases. Random effects linear regression: monthly rate of decline in ALSFRS-R score estimated as −0.58 (95% CI:* −*0.69,* −*0.48) on the summation scale and* −*0.33 (95% CI:* −*0.39,* −*0.27) on the interval scale.*

Figure 1 shows the natural progression of the ALSFRS-R scores across the five visits for the full ProSec3 population. The figure shows a general downward trend both for all data and for the subgroup of individuals for whom ALSFRS-R data were available for five visits, with a random effects linear regression estimate of a 0.58 point monthly rate of decline. Mean ALSFRS-R scores and their standard deviations for each visit are given in Table 2.

**Table 2.** ALSFRS-R outcome data.

ProSec 3 population					
		Summation scale		Interval scale	
Scale	*N*	Mean	SD	Mean	SD
Visit 1 (baseline)	455	30.8	10.1	24.2	6.2
Visit 2 (3 months)	308	29.0	10.6	23.2	6.4
Visit 3 (6 months)	201	29.7	10.2	23.6	6.1
Visit 4 (9 months)	132	29.6	9.7	23.4	6.0
Visit 5 (12 months)	81	27.7	9.8	22.2	5.3
Participants with data at visits 1 and 2					
Visit 1	303	31.1	10.2	24.4	6.2
Visit 2	303	29.1	10.6	23.2	6.3
Those who answers GRoC at visit 2 with ALSFRS-R data at visits 1 and 2					
Visit 1	145	30.2	10.4	23.8	6.2
Visit 2	145	27.5	10.9	22.4	6.5

*Note*: The ALSFRS-R is scored on a 0 (poor) to 48 (good) health scale. NB: due to the GRoC introduction mid-study, there are substantially fewer participants with data for these questions at visits 1 and 2 compared to ALSFRS-R data, which was collected throughout the entire study.

### Anchor-based method

Table 3 presents the mean scores for visits 1 and 2 and the difference in mean for each of the three responses to the GRoC question. The one-way ANOVA for the difference in means across the three categories (“worse,” “about the same,” and “better”) was *p* = 0.025 for the summation scale and *p*=0.077 for the interval scale. The analyses were repeated removing participants with slow-progressing PLS but results were very similar (Supplementary Tables 1 and 2). Spearman’s correlation coefficient was estimated to be 0.2 between the GRoC and the total score of the ALSFRS-R.

**Table 3.** Mean changes in ALSFRS-R score between visits 1 and 2 grouped by GRoC outcome at visit 2.

Summation scale	*n*	Mean Visit 1	Mean Visit 2	Mean difference	SD of mean difference	95% CI for mean difference
Worse	72	29.1	25.3	−3.8	5.2	(−5.0, −2.6)
About the same	69	31.1	29.8	−1.3	5.5	(−2.6, 0.05)
Better	3	30.0	28.3	−1.7	5.5	(−7.9, 4.6)
Interval scale	*n*	Mean Visit 1	Mean Visit 2	Mean difference	SD of mean difference	95% CI for mean difference
Worse	72	22.9	21.0	−2.0	2.8	(−2.6, −1.3)
About the same	69	24.7	23.9	−0.7	3.6	(−1.6, 0.1)
Better	3	23.7	23.4	−0.2	2.4	(−2.9, 2.5)

*Note*: The ALSFRS-R is scored on a 0 (poor) to 48 (good) health scale. A negative mean difference implies health/function declined (got worse) from visit 1 (baseline) to visit 2. ANOVA: *p* = 0.025 for summation scale, *p* = 0.077 for the interval scale.

### Distribution-based methods

Cohen defined a 0.2 standardized effect size as small and 0.5 as medium (15). Using the between-person SD at baseline for the full ProSec3 cohort (*n* = 455), of 10.1 points (summation scale) and 6.2 (interval scale), a small standardized effect size in ALSFRS-R for the summation scale is 2.0 points and a medium 5.0 points (Table 4). For the interval scale a small standardized effect size is 1.2 points and medium is 3.1 points. These results indicate that a mean between-group difference of just 2.0/1.2 points on the ALSFRS-R would represent a small treatment effect. A difference of 5.0/3.1 points on the ALSFRS-R would be considered a medium-sized effect over the 3-month period. Very similar results were seen when the PLS participants were removed (Supplementary Table 3).

**Table 4.** Standardized effect sizes using a baseline SD of 10.1 points (summation scale) and 6.2 points (interval scale).

Standardized effect size	ALSFRS-R (summation scale)	ALSFRS-R (interval scale)
0.2 SD	2.02	1.23
0.3 SD	3.03	1.85
0.4 SD	4.04	2.46
0.5 SD	5.04	3.08

Using the two different estimates of reliability, the SEM was calculated to be 5.43 for internal consistency and 2.25 for test-retest reliability when using the summation scale (Table 5). As the values used to calculate the SEM were from previous studies based on the summation score method, no estimate of SEM has been made for the interval scale.

**Table 5.** Estimates of SEM for the summation scale.

Scale	SD	*r*	SEM = SD × Sqrt(1 − r)
Summation	10.1	0.71 (internal consistency)	5.43
Summation	10.1	0.95 (test - retest)	2.26

**Figure 2.**
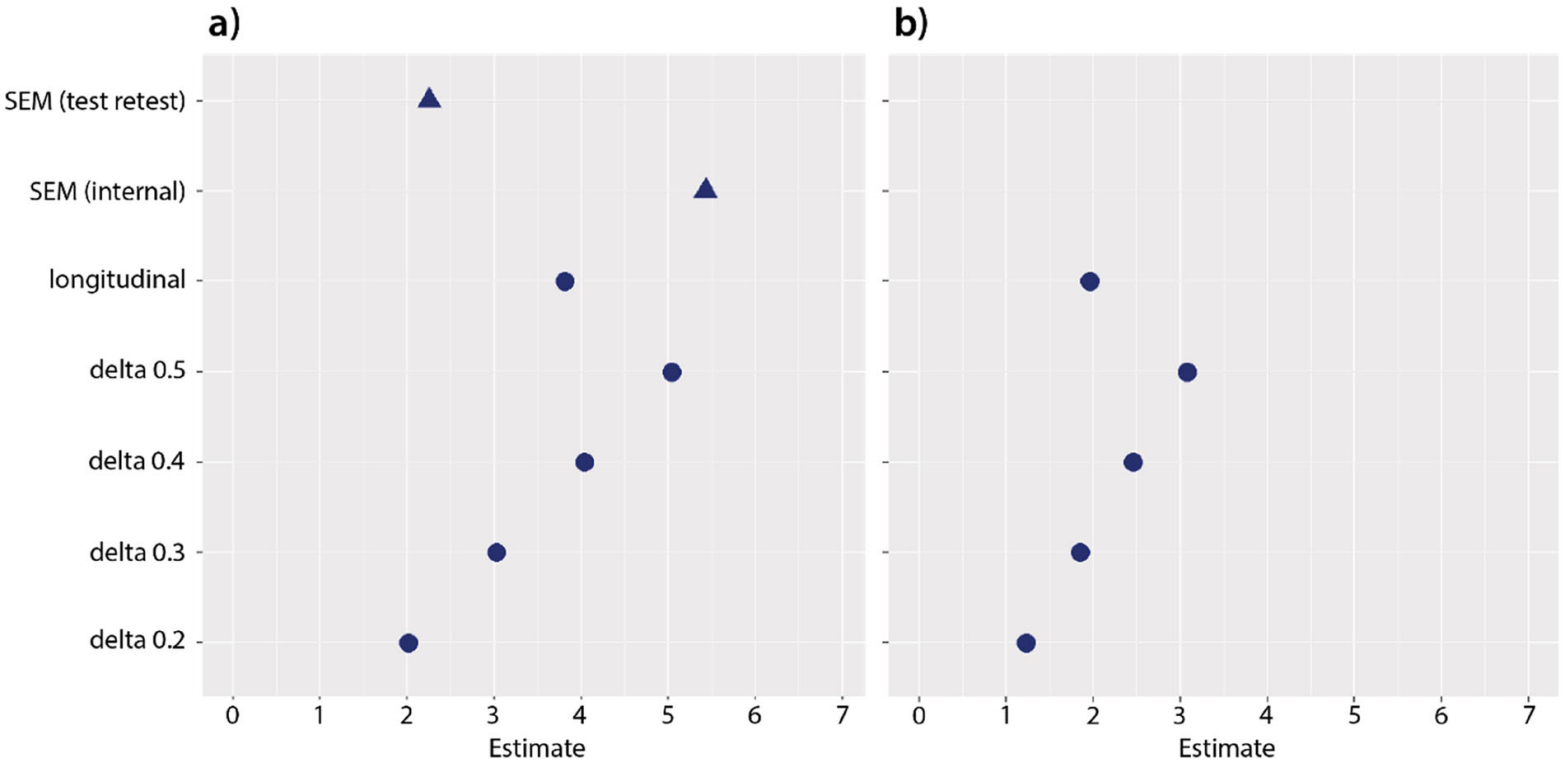
Estimates for the MID at group level (circles) and individual level (triangles) for the ALSFRS-R summation scale (a) and transformed interval scale (b). The y-axis labels are the same for both graphs.

Figure 2 combines the estimates for the MID for both the anchor and distribution based methods. The longitudinal estimate for the MID of 3.8 (summation scale) and 2 (interval scale) are between the small and the medium effect size estimates.

## Discussion

We have used anchor- and distribution-based methods to provide estimates of the MID relating to quality of life for for the ALSFRS-R instrument using a cohort of patients with MND. Longitudinal anchor MID estimates of 3.8 for the summation scale and 2.0 for the interval scale were found to be centered among their respective distribution-based estimates. Hence, we propose an MID for the ALSFRS-R over a 3-month timeframe (as considered in this study) to be a 3.8-point difference between groups when using the summation method of scoring, and 2.0 when scores are transformed using the Young et al. method (11) These estimates translate to 1.27 and 0.67 points per month as a rate of decline over this time period. Based on these findings, we would expect a new drug to out-perform its control by a minimum of 3.8 points over a three month period (1.27 points/month) when comparing summation ALSFRS-R scores or 2.0 points (0.67 points/month) when comparing transformed scores. If the mean of the treatment group is not at least this magnitude higher than that of the control group (reflective of the ALSFRS-R declining with worsening symptoms), then any effect observed should not be considered to have any perceptible health benefit. A treatment not reaching the MID should be scrutinized very heavily with regard to the level of burden associated with its administration. Our study found that the group of participants who did not consider their overall health to have declined between visits were associated with an overall ALSFRS-R decline of 1.3 points (summation scale) and 0.7 points (interval scale) over each 3 month period (0.43 points/month and 0.27 points/month). Therefore, it is crucial to consider quality of life when judging the value of a therapy as side-effects/treatment burden may undo any benefit offered in terms of slowing functional deterioration.

Previous studies have attempted to estimate the MID for the summation-based ALSFRS-R total scores (20) and individual domains (21) with mixed results. The first was based on 81 participants across 5 US sites and failed to find any consistent correlation between the ALSFRS-R domains and quality of life scores (21). As such, no estimate of MID was made for the ALSFRS-R domains. The most recent study conducted at a single site in the US estimated the MID for total ALSFRS-R score change to be 3.24 over approximately 6 months, based on 86 participants who believed their condition to have worsened and 28 who considered their quality of life to have remained the same over that time period (20). As ours and the US study collected ALSFRS-R and quality of life data at different time intervals (3 month and 6 month respectively), both must be converted to a monthly rate of decline to directly compare the findings. The US study-derived MID converts to a monthly rate of decline of approximately 0.54, which is below our estimate of 1.27 points per month. As the ALSFRS-R does not decline in a linear fashion, it may not be appropriate to convert either score to a monthly rate of change and longitudinal data across a wide range of time intervals should be collected to estimate the MID rather than attempting to extrapolate based on a rate-of-change estimate. The Young et al. ALSFRS-R score transformations may overcome this problem and it would be useful for future estimates of MID to consider using this method, as in our current study. It should be noted that in addition to collecting data at different time intervals, our population had a slow rate of disease progression (−0.58 ALSFRS-R points per month (95% CI: −0.68, −0.48) compared to the Fournier study.

The MDC has been previously defined as the smallest change in score (at an individual level) that can be detected after allowing for instrument measurement error. There are several methods for estimating the MDC, usually involving the SEM (17). We estimated the SEM for the summation scale using two methods, the test–retest reliability and internal consistency, yielding estimates of of 2.3 and 5.4. If possible, for a sensitive and reliable instrument, the MDC should be smaller than the MID (17). The MID value estimated for the summation scale from the anchor-based assessment was 3.8 which is smaller than the SEM of 5.4 (internal consistency). In these circumstances, changes as large as the MID may be important for patients, but they cannot be distinguished from measurement error (19). On the other hand, the MID of 3.8 (summation scale) was slightly larger than the SEM of 2.3 (test-retest). In this situation, changes as large as the MID can be considered statistically significant and important to patients (19).

There are a number of limitations of this study and those previously undertaken meaning further research is necessary to narrow the estimated MID range. Our study only involved 145 participants and the standard deviations were relatively high. Future studies should include a larger number of participants and potentially longitudinal data spanning timeframes commonly reported in clinical trials, for example, 6, 12 and 24 months, although evidence suggests longer recall periods are associated with reduced accuracy (22). Indeed, it would also be of interest to consider shorter time intervals (e.g. 4–6 weeks) in a fast-progressing subgroup to detect small yet important declines in ALSFRS-R scores. With a larger cohort, the MID analyses could be separated between fast and slow progressors and potentially between other groupings such as onset site and disease subtype, which we could not investigate satisfactorily due to the very low numbers of non-ALS participants. In clinical trials, efforts are made to predict an individual’s disease trajectory based on criteria such as ALSFRS-R slope to baseline and genetic indicators. It would therefore be of value to collect sufficient data from subgroups to estimate their MIDs independently. As our study cohort’s monthly rate of decline was slower than most clinical trial populations (23), our estimate of MID may not be applicable to fast-progressing subgroups of plwMND. Lastly, on the subject of subgrouping, it may also be worth investigating whether the MID varies at different stages of the disease. As our study population was small and participants were not routinely recruited at time of diagnosis, we could not investigate this with our data.

Another limitation of our study is that the correlation coefficient of the ALSFRS-R and GRoC was only 0.2, below the recommended threshold of 0.3. Therefore, a better anchor should be sought for future studies. There are validated instruments assessing the global quality of life of people with ALS, the ALSSQOL (24) and its curtailed versions, the ALSSQOL-R (25) and ALSSQOL-SF (26). The shortest of these (ALSSQOL-SF) consists of 20 items and is reported to take less than 5 min to complete (26), so would offer a potential alternative anchor for future studies investigating the MID of the ALSFRS-R. A further alternative is the ALSAQ-40, a 40-item health-related quality of life measure for which an MID has been estimated (27,28). It is noteworthy that overall quality of life has been shown not to correlate with physical function in plwMND (29–31), so the more health targeted ALSAQ-40 would offer the possibility to identify health-specific QoL gains.

The use of transition questions or global ratings of change has been widely used to help determine the minimum important difference or change in patient reported outcome measures (PROMS). However, the number of categories in the transition question (we had 3) and the descriptive wording of the question may influence the values reported for the MID. Interviewing participants about the change in their symptoms may have provided more granularity about the extent of the change and this is a limitation. The natural history of MND is for a patient’s functioning and quality of life to decline over time as their disease progresses. There are only three people who scored better on the GRoC questionnaire. Of those three people, two improved their ALSFRS-R score by 1 point and 2 points, respectively. The third person’s score decreased by 8 points. This patient also decreased in all four domains of the ALSFRS-R. It is not clear why this particular respondent reported an improvement on the GRoC. This may be a reflection of the ALSFRS-R questionnaire or how it links function and QoL with one person or the lack of sensitivity of the GroC question. Again, interviewing participants about the change in their symptoms may have provided more granularity about the extent of the change and this is a limitation.

A final limitation of note is that no formal method for imputation of missing ALSFRS-R data exists. This should be rectified, particularly in light of proposed moves toward remote monitoring of study participants alongside self-complete versions of the ALSFRS-R.

The ALSFRS-R itself has limitations regarding its interpretation as a non-linear, multi-dimensional instrument (32). Another measure of function has been developed, the rasch-built overall ALS disability scale (ROADS) (33), which does not share the ALSFRS-R’s limitations and as such may prove a better outcome measure for clinical trials over time. A ROADS MID has already been estimated (20). In addition, inclusion of quality of life measures in clinical trials should be routine to prevent prioritization of life extension at all cost (34).

The results from this study should be considered in future research when determining sample sizes and interpreting ALSFRS-R changes over time between and within groups of people with ALS/MND. Defining the MID is essential to ensure the safeguarding of patients at this time of much welcomed investment in ALS research.

**Table:** 

Affiliation	Name	Email
Sheffield Teaching Hospitals NHS Foundation Trust	Prof Christopher McDermott	c.j.mcdermott@sheffield.ac.uk
Lancashire Teaching Hospitals NHS Foundation Trust	Prof Suresh Kumar Chhetri	suresh.chhetri@nhs.net
Oxford University Hospitals NHS Foundation Trust	Prof Martin R. Turner	martin.turner@ndcn.ox.ac.uk
West Suffolk NHS Foundation Trust	Dr Francesca Crawley	Francesca.Crawley@wsh.nhs.uk
Hywel Dda University Health Board	Mr Joe Annandale	Joe.A.Annandale@wales.nhs.uk
NHS Lothian	Prof Siddharthan Chandran	Siddharthan.Chandran@ed.ac.uk
Northern Care Alliance NHS Foundation Trust	Dr Hisham Hamdalla	Hisham.Hamdalla@nca.nhs.uk
University Hospitals of North Midlands NHS Trust	Dr Thomas Lambert	Thomas.Lambert@uhnm.nhs.uk
Newcastle Hospitals NHS Foundation Trust	Dr Timothy Williams	Tim.Williams@nuth.nhs.uk
University Hospitals Coventry and Warwickshire	Dr Asad Ali	Asad.Ali@uhcw.nhs.uk
St Joseph’s Hospital Auburn	Dr Shea Morrison	Shea.Morrison@svha.org.au
NHS Tayside	Dr Ian Morrison	Ian.Morrison@nhs.scot
The Walton Center NHS Foundation Trust	Prof Carolyn Young	carolyn.young@thewaltoncentre.nhs.uk
Phyllis Tuckwell Hospice Care	Dr Jo Vriens	jo.vriens@pth.org.uk
Sussex Community NHS Foundation Trust	Mrs Jenifer Newton	jenifer.newton@nhs.net
St George’s University Hospitals NHS Foundation Trust	Dr Pablo Garcia-Reitboeck	pgarciareitboeck@nhs.net
King’s College Hospital NHS Foundation Trust	Prof Ammar Al-Chalabi	Ammar.Al-Chalabi@kcl.ac.uk
Cambridge University Hospitals NHS Foundation Trust	Dr Rhys Roberts	rcr20@cam.ac.uk
University Hospitals Plymouth NHS Trust	Prof Oliver Hanemann	oliver.hanemann@plymouth.ac.uk
Keech Hospice Care	Ms Liz Garrood	Formerly Elizabeth.Garrood@keech.org.uk
Medway Community Healthcare	Dr Declan Cawley, Dr Joanna Carrim	declan.cawley1@nhs.net joanna.carrim@nhs.net
LOROS, Hospice care for Leicester, Leicestershire and Rutland	Prof Christina Faull	christinafaull@loros.co.uk
Somerset Partnership NHS Foundation Trust	Ms Suzannah Davies	suzannah.davies@somersetft.nhs.uk
Royal Free London NHS Foundation Trust	Dr Richard Orrell	Formerly r.orrell@ucl.ac.uk
Leeds Community Healthcare NHS Trust	Prof Mike Bennett (St Gemma’s Hospice site), Dr Chris Kane (Wheatfields Hospice site)	mike.bennett3@ntlworld.com (formerly m.i.bennett@leeds.ac.uk) formerly Christopher.kane@nhs.net
St Margaret’s Somerset Hospice	Dr Kate Shorthose	Kate.Shorthose@st-margarets-hospice.org.uk
Manchester University NHS Foundation Trust	Dr Tim Felton, Ms Lara Dickinson (till Sept 2019)	Tim.Felton@manchester.ac.uk lara.dickinson3@nhs.net
Birmingham Hospice (formerly St Mary’s Hospice)	Mrs Ruth Roberts	ruth.roberts@birminghamhospice.org.uk
East Kent Hospitals University NHS Foundation Trust	Ms Christine Batts	christine.batts@nhs.net
Mary Stevens Hospice	Mr Andrew Kenwrick	andrew.kenwrick@marystevenshospice.co.uk
Marie Curie Hospice, Bradford	Dr Gemma Clarke	gemma.clarke@mariecurie.org.uk
Marie Curie Hospice, Solihull	Mr John MacArtney	John.MacArtney@mariecurie.org.uk
NHS Ayrshire and Arran	Dr Jenny Preston	Jenny.Preston@aapct.scot.nhs.uk

## Supplementary Material

Supplementary data.docx
