# Supplementary material for: Estimating the minimum important difference in the ALSFRS-R-instrument in people living with MND
Source: Amyotroph Lateral Scler Frontotemporal Degener. 2025 Feb 3;26(3-4):249–58. doi: 10.1080/21678421.2024.2447916 (PMC12011019; doi:10.1080/21678421.2024.2447916)
Supplement: Supplementary data.docx [file IAFD_A_2447916_SM0215.docx]

| **ProSec 3 Population** | | | | | |
| --- | --- | --- | --- | --- | --- |
| **Scale** |  | **Summation scale** | | **Interval scale** | |
|  | **N** | **Mean** | **SD** | **Mean** | **SD** |
| Visit 1 (baseline) | 413 | 31.0 | 10.1 | 24.4 | 6.2 |
| Visit 2 (3 months) | 276 | 28.9 | 10.9 | 23.3 | 6.6 |
| Visit 3 (6 months) | 180 | 29.6 | 10.5 | 23.6 | 6.3 |
| Visit 4 (9 months) | 116 | 29.3 | 10.1 | 23.2 | 6.2 |
| Visit 5 (12 months) | 72 | 27.2 | 9.9 | 21.9 | 5.3 |
| **Participants with data at visits 1 and 2** | | | | | |
| Visit 1 | 272 | 31.1 | 10.4 | 24.5 | 6.3 |
| Visit 2 | 272 | 29.0 | 10.8 | 23.2 | 6.5 |
| **Those who answers GRoC at visit 2 with ALSFRS-R data at visits 1 and 2** | | | | | |
| Visit 1 | 130 | 30.4 | 10.7 | 24.1 | 6.4 |
| Visit 2 | 130 | 27.6 | 11.3 | 22.6 | 6.8 |

Table 1. ALSFRS-R outcome data after participants with PLS removed. *The ALSFRS-R is scored on a 0 (poor) to 48 (good) health scale. NB: due to the GRoC introduction mid-study, there are substantially fewer participants with data for these questions at visits 1 and 2 compared to ALSFRS-R data, which was collected throughout the entire study.*

| **Summation scale** | **n** | **Mean Visit 1** | **Mean Visit 2** | **Mean difference** | **SD of mean difference** | **95% CI for mean difference** |
| --- | --- | --- | --- | --- | --- | --- |
| Worse | 62 | 29.6 | 25.4 | -4.2 | 5.3 | (-5.5, -2.9) |
| About the same | 64 | 31.2 | 29.8 | -1.4 | 5.7 | (-2.8, 0.02) |
| Better | 3 | 30.0 | 28.3 | -1.7 | 5.5 | (-7.9, 4.6) |
| **Interval scale** | **n** | **Mean Visit 1** | **Mean Visit 2** | **Mean difference** | **SD of mean difference** | **95% CI for mean difference** |
| Worse | 62 | 23.3 | 21.1 | -2.2 | 2.8 | (-2.9, -1.5) |
| About the same | 64 | 24.8 | 24.0 | -0.8 | 3.7 | (-1.7, 0.2) |
| Better | 3 | 23.7 | 23.4 | -0.2 | 2.4 | (-2.9, 2.5) |

Table 2. *ALSFRS-R data for different GRoC groups after participants with PLS removed.* *ANOVA: p=0.017 for summation scale, p=0.051 for the interval scale. The ALSFRS-R is scored on a 0 (poor) to 48 (good) health scale. A negative mean difference implies health/function declined (got worse) from visit 1 (baseline) to visit 2.*

| **Standardised effect size** | **ALSFRS-R (summation scale)** | **ALSFRS-R (interval scale)** |
| --- | --- | --- |
| 0.2 SD | 2.03 | 1.24 |
| 0.3 SD | 3.04 | 1.87 |
| 0.4 SD | 4.05 | 2.49 |
| 0.5 SD | 5.06 | 3.11 |

Table 3: *Standardised effect sizes using a baseline SD of 10.1 points (summation scale) and 6.2 points (interval scale) after data for participants with PLS removed.*
